# Economic evaluation of school scoliosis screening in Gannan Tibetan autonomous prefecture, Gansu Province: a cost-utility analysis based on decision tree-Markov model

**DOI:** 10.3389/fpubh.2026.1801371

**Published:** 2026-05-12

**Authors:** Shaobo Yang, Juan Wang, Peiji Miao, Chen Zhang, Jin Huang, Xiaoyun Yuan, Yanxiang Zhang, Xiaohui Dou, Zhenheng Zhang, Zhe Liu, Jianjun Duan, Xueting Xu, Jiantao Wen, Shunjun Cui, Xiaole Zhu

**Affiliations:** 1 School of Integrative Medicine, Gansu University of Chinese Medicine, Lanzhou, Gansu, China; 2 Department of Spinal Deformity Surgery, Gansu Provincial Hospital of Traditional Chinese Medicine, Lanzhou, Gansu, China; 3 School of Public Health, Gansu University of Chinese Medicine, Lanzhou, Gansu, China; 4 School of Clinical Medicine of Traditional Chinese Medicine, Gansu University of Chinese Medicine, Lanzhou, Gansu, China; 5 School of Public Health, Soochow University, Suzhou, Jiangsu, China; 6 Department of Orthopedics, People’s Hospital of Yongjing County, Linxia, Gansu, China

**Keywords:** adolescent idiopathic scoliosis, cost-utility, Markov model, school-based screening, sensitivity analysis

## Abstract

**Objective:**

This study evaluates the cost-utility of school-based scoliosis screening for adolescents in resource-limited settings to inform regional school health and disease control policies.

**Methods:**

We performed a model-based cost-utility analysis using real-world data from a screening program in Gannan Tibetan Autonomous Prefecture, Gansu Province. A decision tree linked to a Markov cohort model compared “organized school-based screening” with “no organized screening, relying on opportunistic detection.” The model simulated a cohort of in-school adolescents until skeletal maturity over a 4-year baseline horizon, using 3-month Markov cycles. Health states were stratified by adolescent idiopathic scoliosis severity: mild, moderate (with or without bracing), severe (awaiting surgery), and post-operative stable. Adopting a healthcare system perspective, we estimated costs and quality-adjusted life years (QALYs), applying a 5% annual discount rate. Parameter uncertainty was assessed via deterministic and probabilistic sensitivity analyses.

**Results:**

In the base-case analysis, the screening strategy dominated the opportunistic detection strategy, yielding a lower discounted per-person cost and a minimal discounted QALY gain of 0.000005. The incremental net monetary benefit was 58.33 CNY per person at a willingness-to-pay threshold of 52,825 CNY/QALY. Probabilistic sensitivity analysis showed most iterations in the cost-saving quadrant of the cost-utility plane, with incremental QALYs near zero, confirming the strategy’s robust cost-saving potential across parameter uncertainties. The results were most sensitive to the assumed proportion of undiagnosed cases without screening, followed by surgical costs and disease progression probability. Scenario analysis indicated a more pronounced cost-saving advantage in regions with weaker routine diagnostic capacity.

**Conclusion:**

From a healthcare system perspective, school-based adolescent scoliosis screening in resource-limited settings can be cost-saving during adolescence. The primary economic benefit arises from reducing progression to severe disease and avoiding high-cost surgeries. The limited modeled QALY gain likely reflects the short analytical horizon, which inadequately captures potential long-term quality-of-life benefits related to body image, mental health, and functional outcomes in adulthood.

## Introduction

Adolescent idiopathic scoliosis (AIS) is a prevalent structural spinal deformity that develops during adolescence. Because curve progression correlates strongly with residual growth potential, the spinal curvature can deteriorate to a clinically significant degree within a relatively brief period during the adolescent growth spurt (1–3). Upon reaching a moderate or severe threshold, the condition can cause trunk deformity, pain, and psychosocial distress, often necessitating long-term bracing or surgical intervention, which imposes sustained health and economic burdens on individuals, families, and healthcare systems (4–6).

As Adolescent idiopathic scoliosis frequently presents without obvious symptoms in its early stages, passive, presentation-based detection often leads to delayed diagnosis and a missed opportunity for non-surgical management. Consequently, systematic school-based screening is regarded as a potential strategy for early detection (7, 8). Similar to other population-based screening programs, however, the implementation of school-based AIS screening has long been debated across different countries and regions. The central question is not whether screening can identify cases, but whether its population-level implementation delivers sufficient health benefits to justify the associated costs (9, 10).

Previous research has primarily examined the detection rate and clinical effectiveness of AIS screening, while evidence for its economic justification remains limited, particularly in low- and middle-income regions. Existing economic evaluations often rely on hypothetical cohorts or simplified decision models that do not adequately capture the time-dependent progression of AIS across severity states, nor do they incorporate real-world operational parameters such as screening uptake, diagnostic confirmation rates, and local costs. Consequently, their findings may have limited generalizability to resource-constrained settings where healthcare access and diagnostic capacity differ substantially from high-resource environments (11, 12). In certain sparsely populated areas of Northwest China, constraints such as access to specialized care and the timeliness of radiological confirmation may mean the marginal benefit of organized school-based screening is greater than in high-resource environments. Nonetheless, localized economic evidence derived from real-world implementation data to directly inform policy remains scarce.

Within this context, this study evaluates the school-based AIS screening program in Gannan Tibetan Autonomous Prefecture. By quantifying the costs and QALYs of school-based screening versus opportunistic detection under real-world resource constraints, and incorporating sensitivity analyses to address uncertainty, we aim to assess the economic value of this strategy. The findings will provide evidence-based support for school health policy and AIS prevention in underdeveloped regions.

## Methods

### Research design and analysis framework

This study conducted a model-based cost–utility analysis using real-world screening program data to evaluate the economic value of school-based adolescent idiopathic scoliosis (AIS) screening. The school-based screening program was conducted from December 2024 to April 2025. A decision-analytic approach was adopted, constructing a decision tree combined with a Markov cohort model to compare two strategies: implementing organized school-based AIS screening versus no organized screening, relying solely on passive clinical presentation or opportunistic detection. This study adheres to the CHEERS 2022 reporting checklist (13).

### Study population and time horizon

The model simulated a cohort of school-going adolescents, consistent with the actual target population of school-based screening programs. All individuals were assumed to be in the initial screening state upon model entry and were followed in the model until skeletal maturity. In the base-case scenario, the follow-up time was set at 4 years to cover the critical growth period for the occurrence and progression of AIS (14).

The Markov cycle length was set at 3 months to reflect the potential progression characteristics of scoliosis during adolescent growth spurts and to align with the commonly used 3–6 month interval for clinical follow-up/radiographic reassessment (15, 16). Both costs and health utilities in the model were discounted at an annual rate of 5%, in accordance with the recommendations of the *Chinese Guidelines for Pharmacoeconomic Evaluations* (17).

### Model parameter settings and data sources

Model parameters included screening and epidemiological parameters, disease natural history transition probabilities, intervention effect parameters, health utility values, and cost parameters. Data on screening scale, suspected positive rate, compliance rate for confirmation, number of confirmed cases, and severity distribution were derived from real-world school-based screening program data in Gannan Tibetan Autonomous Prefecture. The baseline values, ranges, uncertainty distributions, and data sources for the relevant parameters are detailed in Tables 1–6.

**Table 1:** Model structure parameters.

Parameter category	Parameter name	Baseline value	Value range/uncertainty	Distribution type	Data source
Model type	Decision Tree + Markov state transition model	—	—	—	ISPOR-SMDM modeling good research practices (22)
Research perspective	Health system perspective	—	—	—	China guidelines for pharmacoeconomic evaluations (2020) (17)
Cycle length	3 months/cycle	—	—	—	AIS follow-up frequency (23).
Time range	Until skeletal maturity (average 4 years, 16 cycles)	2–5 years	Scenario analysis	—	Growth and development period of AIS and observation window (23).
Severity threshold	Cobb angle ≥45°	Fixed	—	—	SRS/SOSORT guidelines (8)
Discount rate (cost and utility)	5% per annum	0–8%	Fixed	China guidelines for pharmacoeconomic evaluations (2020) (17)	—
Willingness-to-pay threshold (WTP)	52,825CNY/QALY (1 × GDP)	1–3 × GDP	Scenario analysis	Per capita GDP of Gansu Province in 2024	Gansu provincial bureau of statistics

**Table 2:** Epidemiology and screening parameters.

Parameter category	Parameter name	Baseline value	Value range/uncertainty	Distribution type	Data source
Total number of people screened	52,678	Fixed	—	—	On-site data
Number of suspected positive cases	1,211	Fixed	—	—	On-site data
Suspected positive rate	0.0230	±20%	Beta	On-site data calculation.	—
Completion rate of X-ray confirmation.	0.277	±20%		336/1211	—
Number of confirmed AIS Cases	172	Fixed	—	—	On-site data
AIS detection rate.	0.00326	±20%	Beta	172/52,678	—
Diagnosis stratification (Mild/Moderate/Severe)	118/41/13	±20%	Dirichlet	On-site data	—
Brace initiation rate after moderate diagnosis	0.902	±20%	Beta	On-site data (37/41)	—

**Table 3:** Natural history and transition probabilities (3-month cycle).

Parameter category	Parameter name	Baseline value	Value range/ uncertainty	Distribution type
Mild to moderate	0.016	±50%	Calibration	Literature anchor points + calibration (24, 25)
Moderate to severe (without Orthotic)	0.083	±50%	Derive	BrAIST observation group (26)
Hazard ratio (HR) of Orthotic	0.52	0.30–0.80	Log-normal	BrAIST trial (26)
Moderate to severe (without Orthotic)	0.045	±50%	Derive	Calculated as “without Orthotic adjusted transition probability × HR/azard ratio” (constant risk/rate-probability conversion methodology)
Severe to surgery (within 1 year)	0.90	0.70–0.98	Beta	Literature-based (39) + Local Waiting Area
Severe to awaiting surgery (per cycle).	0.206	Derive	—	Constant Risk Conversion: Rate ↔ Probability

**Table 4:** Utility values.

Parameter category	Parameter name	Baseline value	Value range/uncertainty	Distribution type
Mild AIS	0.96	±0.05	Beta	Study on the Reliability and Validity of EQ-5D-5L in Chinese AIS Population (28)
Moderate AIS (without Orthotic)	0.93	±0.05	Beta	Study on the Reliability and Validity of EQ-5D-5L in Chinese AIS Population (28)
Moderate AIS (Orthotic)	0.89	±0.05	Beta	SRS-22r to EQ-5D-5L mapping study (29)
Severe AIS.	0.91	±0.05	Beta	Mapping/extrapolation + sensitivity analysis coverage (29)
Postoperative status	0.86	±0.05	Beta	Mapping/extrapolation + sensitivity analysis coverage (29)

**Table 5:** Cost parameters (US$).

Parameter category	Parameter cost (US$)	Baseline value	Value range/uncertainty	Distribution type
Total cost of screening organization	9,269.06	±20%	Gamma	On-site data
Per capita screening cost	0.18	Derive	—	9,269.06/52,678
X-ray confirmation cost	50.54/per time	±20%	Gamma	Local charges
Orthotic cost	772.20/capita	±20%	Gamma	On-site data
Follow-up costs (Universal Screening Arm)	52.51/cycle	±20%	Gamma	On-site data
Follow-up costs (No Active Screening Control Arm)	26.25/cycle	±20%	Gamma	Twice per year
Surgical expenses	1965.60/capita	±20%	Gamma	On-site data

**Table 6:** No active screening control arm (calibration parameters).

Parameter category	Parameter name	Baseline value	Value range/uncertainty	Distribution type
Missed diagnosis rate θ_miss.	0.30	0.15–0.50	Beta	Literature anchor points + calibration (25, 30)
Initial diagnosis distribution (mild/moderate/severe).	0.235/0.521/0.244	Scenario A/B.	Dirichlet	Literature anchor points (30)

For parameters available in the literature, published data were used directly. When published data did not align with the model’s three-month cycle length, a constant risk assumption was applied to convert annual or long-term probabilities into cycle-specific transition probabilities (18, 19). For key parameters lacking direct local evidence, values were derived by integrating literature-based anchor points with model calibration, with their uncertainty fully addressed in sensitivity analyses (20, 21).

### Screening pathway and decision tree structure

**Figure 1:**
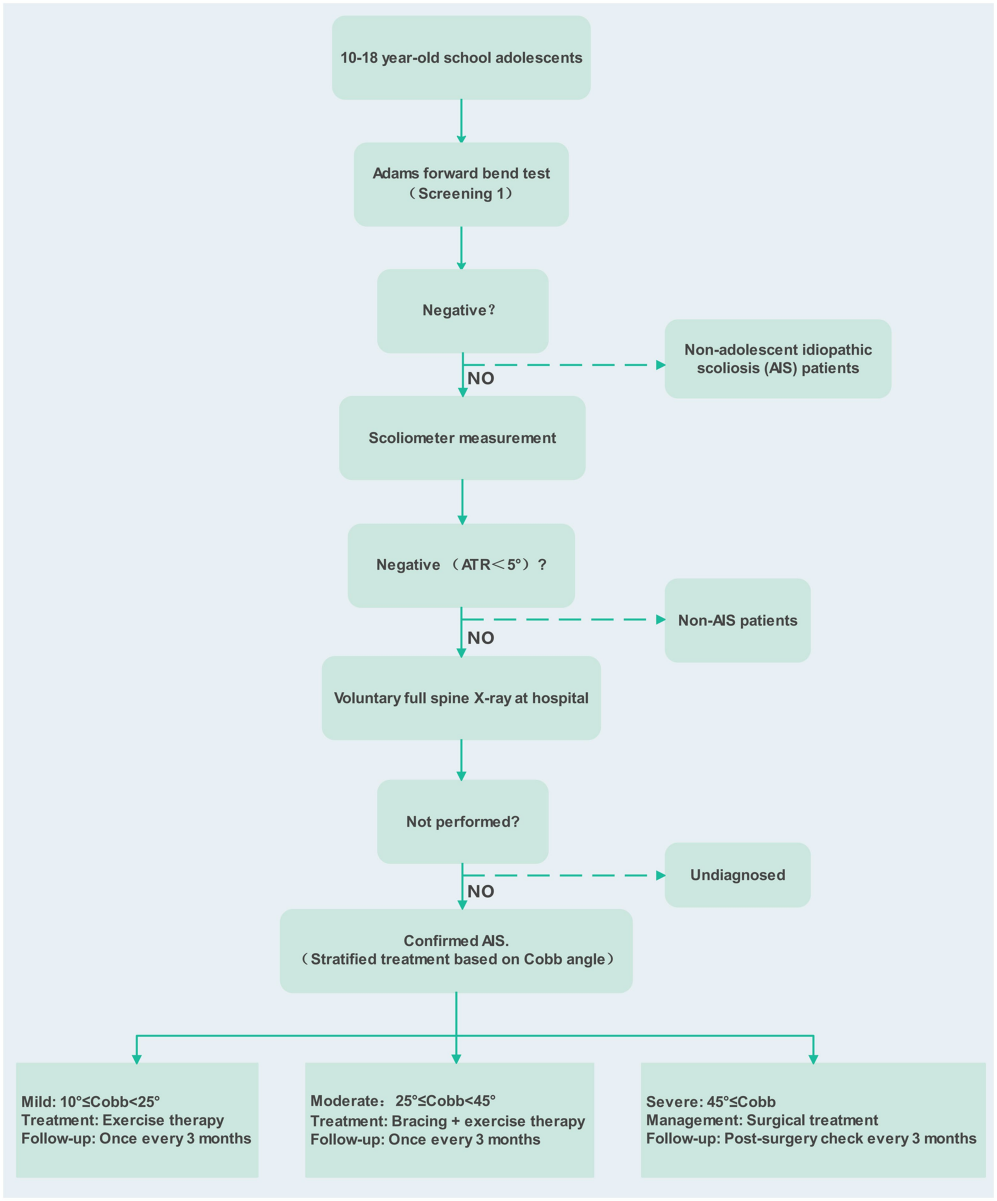
School entry screening flowchart.

The school-based screening strategy was modeled as a comprehensive continuum, comprising initial screening, secondary screening, radiological confirmation, and subsequent clinical management. The process began with the Adams forward bend test for primary screening, followed by an electronic scoliosis measurement device for secondary screening. Individuals testing positive in the secondary screening were classified as suspected AIS and could voluntarily undergo a full-spine X-ray examination. They were then stratified into corresponding management pathways based on Cobb angle severity (5) (Figure 1).

In the model, individuals with suspected positivity who did not complete radiological confirmation were assumed to derive no long-term health benefits from screening, with their disease progression trajectory mirroring that of the unscreened population. This conservative assumption, which aligns with prior economic evaluations of population screening, aims to avoid overestimating screening benefits by accounting for those who do not complete diagnostic confirmation (20, 31–33). Under the no-screening strategy, individuals were only detected and diagnosed opportunistically when postural abnormalities became apparent, subjective symptoms emerged, or indications were found during physical examinations. In the no-screening arm, no school-based scoliosis screening was implemented, so diagnosis occurred solely through opportunistic discovery, such as during school physical examinations, when postural abnormalities were noticed by the individual or others, or when significant deformity was present.

### Markov model structure

**Figure 2:**
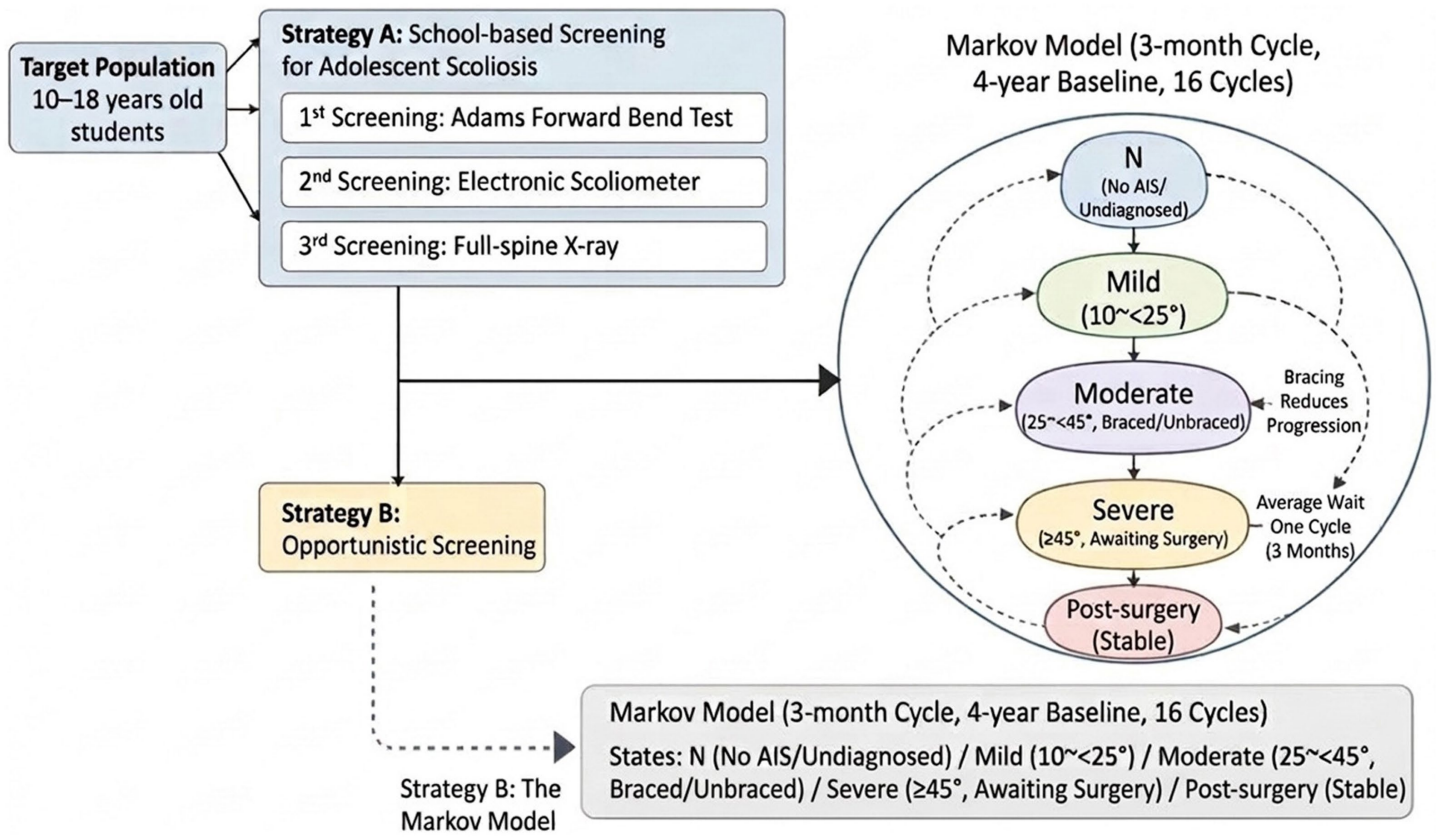
Schematic diagram of the decision tree-Markov model structure for adolescent idiopathic scoliosis under school entry screening and opportunistic screening strategies.

Individuals diagnosed with AIS entered a Markov state-transition model to characterize the long-term natural history of the disease during growth and the impact of interventions. The model included the following health states: No AIS/Undiagnosed (N), Mild AIS (10° ≤ Cobb < 25°), Moderate AIS (25° ≤ Cobb < 45°, subdivided into brace-treated and non-brace-treated), Severe AIS (Cobb ≥ 45°, awaiting surgery), and Postoperative Stable State (32, 33). The model allowed for disease progression or treatment receipt within each cycle. Patients with Severe AIS were modeled to undergo surgery after an average waiting period of at least one cycle, subsequently transitioning to the Postoperative Stable State. A schematic of the model structure is presented in Figure 2 (5).

### Transition probabilities and intervention effects

Baseline transition probabilities for AIS were obtained from longitudinal natural history studies. For moderate AIS, the effect of brace treatment was applied as a hazard ratio to the probability of progression from moderate to severe. This method aligns with prior clinical and economic evaluations (7, 18, 19).

The model assumed the treatment effect persisted throughout the wearing period, without dynamically modeling changes in compliance over time. Parameter uncertainty was evaluated via one-way and probabilistic sensitivity analyses (20, 34).

### Costs, utilities, and discounting

Costs, measured in Chinese Yuan (CNY), encompassed screening organization, diagnostic imaging, braces, follow-up, and surgery, based on local charges or study assumptions. To enable international comparison and account for temporal variations in exchange rates, according to data from the National Bureau of Statistics of China all costs originally recorded in CNY were converted to US dollars (US$) using year-specific average exchange rates: 2024 (1 CNY = 0.1404 US$) (35). Health outcomes were measured in QALYs. Utility values for each health state, derived from previous EQ-5D studies, were held constant within each Markov cycle (28, 36).

The analysis adopted a healthcare system perspective, excluding indirect and patient-level non-medical costs. The results therefore provide a conservative estimate of the economic value of school-based screening (17).

### Literature anchor points and calibration without screening arm parameters

Given the lack of direct observational data on the distribution of initial diagnosis severity and the proportion of underdiagnosed cases without school-based screening, this study constructed a no-screening control scenario using a “literature anchor + calibration” approach. First, international studies reporting the distribution of initial AIS diagnoses in settings without school screening were selected as external anchors, including data from Johnson et al. (24) and Hoelen et al. (30), and mapped to the model’s defined severity thresholds (mild: Cobb angle 10° ≤ Cobb < 25°, moderate: 25° ≤ Cobb < 45°, severe: Cobb ≥ 45°). Free parameters were then introduced to calibrate both the initial diagnosis distribution and the underdiagnosis proportion (θ_miss). The calibration process aimed to match the literature-based distribution of initial diagnoses and the assumed underdiagnosis rate. The fitting criteria adopted a maximum likelihood estimation method, with parameter uncertainty evaluated via beta distribution for θ_miss (range: 0.15–0.50) and Dirichlet distribution for the initial diagnosis distribution. The calibration was validated by comparing the simulated no-screening outcomes with real-world opportunistic detection data from Gannan Prefecture, such as the baseline detection rate of 0.326%. To test the robustness of the conclusion, two scenario analyses were conducted: Scenario A (θ_miss = 0.15, representing a region with strong routine diagnostic capacity) and Scenario B (θ_miss = 0.50, representing a region with weak diagnostic capacity). The model’s transparency and validity were assessed following the ISPOR-SMDM recommendations on transparency and validation (21).

### Sensitivity analysis

A one-way sensitivity analysis (OWSA) was performed on all key model parameters, with the incremental net monetary benefit (INMB) serving as the evaluation metric. The results are presented using Tornado diagrams (34, 37). Probabilistic sensitivity analysis (PSA) was also conducted via Monte Carlo simulation to evaluate the impact of joint parameter uncertainty on the model’s conclusions. The parameter distribution settings and assumptions align with those presented in the table (20, 34).

## Results

### Baseline implementation and detection results of the school-based screening program

A total of 52,678 students from Gannan Prefecture were enrolled in this school-based screening study. Following the secondary screening, 1,211 individuals were identified as suspected cases of Adolescent Idiopathic Scoliosis (AIS), yielding a suspected positive rate of 2.30% (1,211/52,678). Among these suspected cases, 336 individuals completed full-spine X-ray examinations for definitive diagnosis, resulting in a compliance rate from suspicion to confirmation of 27.7% (336/1,211). Ultimately, 172 cases were confirmed as AIS, corresponding to an overall detection rate of 0.326% (172/52,678). Among those who completed X-ray examinations, the positive predictive value (PPV) of X-ray (confirmed AIS / X-ray performed) was 51.2% (172/336). When using “positive results from both screening stages” as the suspicion criterion, the PPV of the two-stage screening (confirmed AIS / suspected) was 14.2% (172/1,211). The severity distribution among confirmed cases was as follows: 118 mild cases, 41 moderate cases, and 13 severe cases. The total organizational cost of the screening program was 66,019 Chinese Yuan (CNY), translating to a screening cost of 1.25 CNY per person (66,019/52,678), which falls within the lower range of previously reported costs for school-based screenings (Table 7) (9).

**Table 7:** Baseline data in screening.

Factors	Value
Number of screenings.	52,678
Presumptive positive (Double-test positive).	1,211
Suspected positive rate.	0.0230
Complete X-ray confirmation.	336
Suspected→Confirmed Compliance Rate.	0.277
Diagnosed with AIS.	172
AIS detection rate (Confirmed cases/screened cases).	0.003
X-ray PPV (Confirmed AIS/films taken).	0.5112
Double-check PPV (confirmed AIS/suspected).	0.1420
Mild/moderate/severe (Person).	118/41/13
Screening cost per person (US$).	0.1760

### Composition of model input parameters and baseline settings

Model parameters comprised screening and epidemiological inputs, natural history transition probabilities, intervention effects, utility values, and costs. The screening scale, suspected case rate, confirmed diagnosis compliance rate, number of confirmed cases, and severity distribution were all obtained from the real-world screening data in this study (52,678 individuals; 1,211 suspected cases; a compliance rate of 0.277; 172 confirmed cases; mild/moderate/severe = 118/41/13). The brace initiation rate following a moderate diagnosis was 0.902 (37/41). Key economic assumptions adopted a healthcare system perspective, a 3-month cycle, and a time horizon extending to skeletal maturity (baseline: 4 years, 16 cycles). Costs and utilities were discounted at 5% annually, with the willingness-to-pay (WTP) threshold set at 52,825 CNY/QALY, equivalent to one times the per capita GDP (Table 8).

**Table 8:** Model input parameters (AISversion).

Parameter	Base-case value	Distribution	Range / uncertainty	Data source / notes
Cohort size (students screened)	52,678	Fixed	—	Screening data
Suspected positives (positive on two-stage screening)	1,211	Fixed	—	Screening data
Proportion of suspected cases completing radiographic confirmation	336/1,211 = 0.2775	Beta	±20%	Screening data
Confirmed AIS cases	172	Fixed	—	Screening data
Severity distribution at diagnosis (mild / moderate / severe)	118/41 / 13	Dirichlet	±20%	Screening data
Total screening cost (US$)	9,269.0676	Gamma	±20%	Screening expenses (meals + accommodation + transportation)
Radiographic confirmation cost (US$ per exam)	50.544	Gamma	±20%	Local fee schedule
Brace cost (US$ per patient, one-time)	772.2	Gamma	±20%	Local average brace cost (study assumption)
Bracing initiation rate after moderate AIS diagnosis	0.90244	Beta	±20%	Screening data
Follow-up cost (screening arm, US$ per cycle)	52.5096	Gamma	±20%	374 CNY per visit; one visit every 3 months (cycle length = 3 months)
Follow-up cost (no-screening arm, US$ per cycle)	26.2548	Gamma	±20%	Two visits per year → 0.5 visit per cycle; 374 × 0.5 = 187
Surgical cost (US$ per case)	19,656	Gamma	±20%	Study assumption
Probability of surgery within 1 year among severe cases (≥45°)	0.90	Beta	0.70–0.98	Study assumption
Severe → surgery-waiting (per 3-month cycle)	0.4377	Derived	—	Converted from 0.90/year assuming a constant hazard
Moderate → severe progression without bracing (per 3-month cycle)	0.0877	Derived	±50%	Derived under a constant-hazard assumption (7)
Bracing effect (HR_brace)	0.45	Lognormal	0.30–0.80	Approximated as a hazard ratio under a constant-hazard assumption (7, 19)
Moderate → severe progression with bracing (per 3-month cycle)	0.0405	Derived	—	Derived using HR_brace and the non-bracing hazard under a constant-hazard assumption (19)
Mild → moderate progression (per 3-month cycle)	0.0164	Calibrated	±50%	Calibrated/mapped from literature; converted to a 3-month probability assuming a constant hazard (19, 38)
Utility: mild AIS	0.95	Beta	0.90–0.99	Literature-based (49)
Utility: moderate AIS without bracing	0.95	Beta	0.90–0.99	Literature-based (49)
Utility: moderate AIS with bracing	0.87	Beta	0.80–0.93	Literature-based (39)
Utility: severe AIS	0.76	Beta	0.65–0.82	Literature-based (40)
Utility: post-surgery (stable)	0.82	Beta	0.75–0.90	Literature-based (40)
Discount rate (costs and utilities)	0.05	Fixed	0–0.08 (sensitivity)	China Guidelines for Pharmacoeconomic Evaluations (2020) (17)
Willingness-to-pay (WTP) threshold	7,416.63 US$/QALY (1 × GDP per capita)	Fixed	1–3 × GDP per capita	Gansu Statistical Bulletin 2024: GDP per capita
Missed-diagnosis proportion in the no-screening arm (θ_miss)	0.30	Beta	0.15–0.50 (scenario)	Modeled as a calibration/scenario parameter; explored in sensitivity analyses (41, 42)
Severity distribution at first presentation in the no-screening arm (mild / moderate / severe)	0.235/0.521/0.244	Dirichlet	Scenario A: 0.15/0.50/0.35; Scenario B: 0.35/0.50/0.15	Literature-informed and mapped/calibrated (30)

AIS, adolescent idiopathic scoliosis; HR, hazard ratio; QALY, quality-adjusted life year; WTP, willingness-to-pay; GDP, gross domestic product; CNY, Chinese yuan. Cycle length = 3 months. “Derived” indicates conversion from annual risks or cumulative incidences to cycle-specific probabilities assuming a constant hazard; “Calibrated” indicates parameters estimated via calibration to reproduce external targets.

### Cost-utility analysis results in the baseline scenario

At a willingness-to-pay (WTP) threshold of 52,825 CNY/QALY, the school-based universal screening strategy incurred a discounted total cost of $18.03 per person and yielded 3.655057 discounted QALYs. The no-screening (opportunistic detection) strategy had a discounted total cost of US$26.18 per person and yielded 3.655052 discounted QALYs. Relative to no screening, universal screening produced an incremental cost of -US$2.32 per person and an incremental QALY gain of 0.000005, resulting in an INMB of US$8.19 per person. Consequently, in the baseline scenario, the universal screening strategy was dominant, as it entailed lower costs and marginally higher QALYs (Table 9).

**Table 9:** Baseline cost-utility results (USD/ per person, discounted).

Strategy	Cost (US$/person, discounted)	QALY (discounted)	Net monetary benefit US$ (US$/person, WTP = 7416.63^*^)
Universal screening arm	18.03	3.655057	27090.17
No active screening control arm	26.18	3.655052	27081.98
Increment	−8.15	0.000005	8.19

Baseline analysis indicated that universal screening was the dominant strategy (lower cost with slightly higher QALY): ΔCost = −8.15 US$ /person, ΔQALY = 0.000005; INMB = 8.19 US$ person (WTP = 7416.63).*Note: The willingness-to-pay threshold of 7,416.63 US$ corresponds to 52,825 CNY, based on the 2024 average exchange rate (1 CNY = 0.1404 US$).

### One-way sensitivity analysis

**Figure 3:**
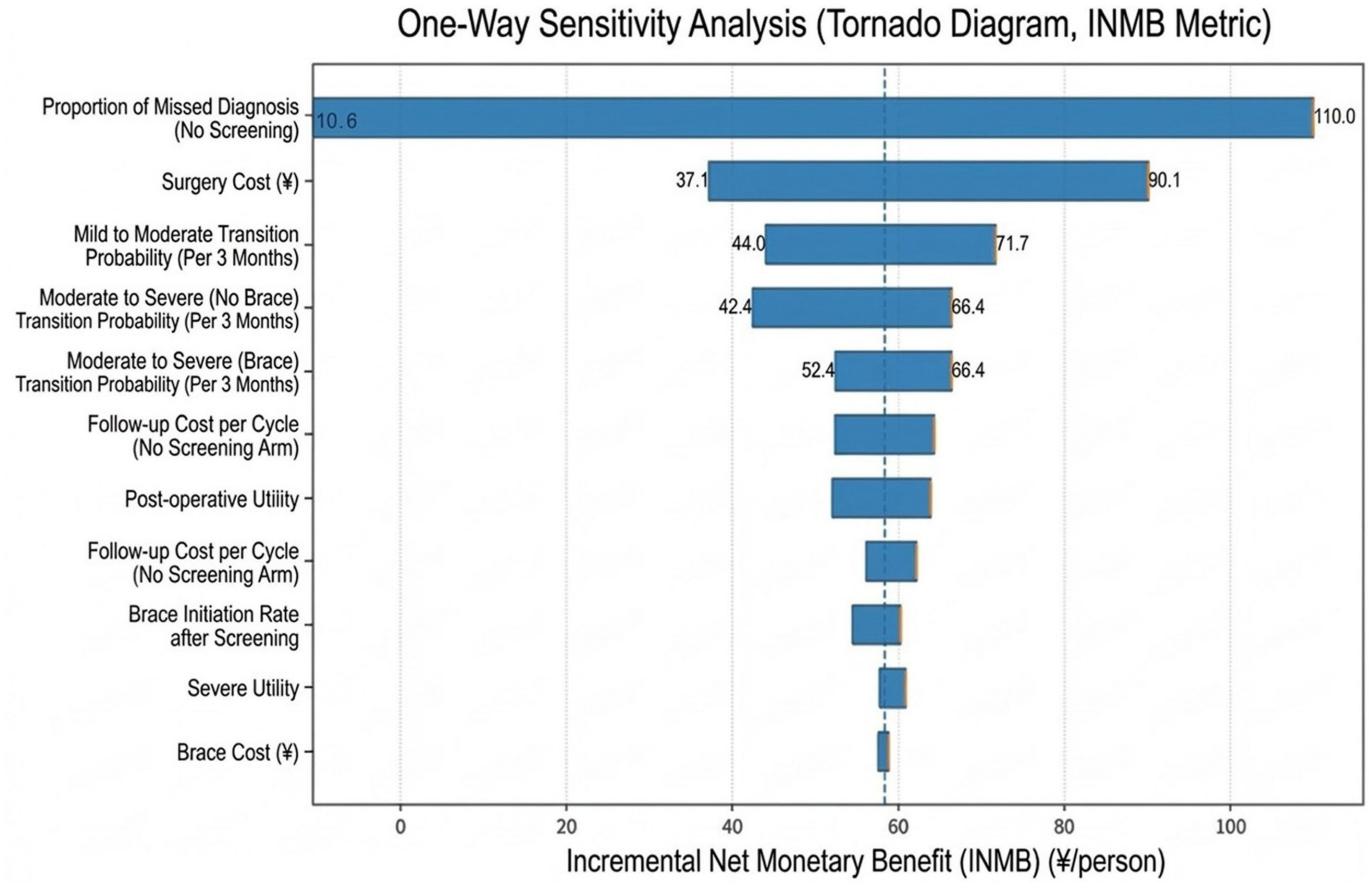
Univariate sensitivity analysis (Tornado diagram, using INMB as the indicator).

The results of the one-way sensitivity analysis, using the incremental net monetary benefit as the outcome measure, are presented in a Tornado diagram (Figure 3). The analysis revealed that the model conclusion was most sensitive to the proportion of underdiagnosed cases in the no-screening arm (θ_miss). Variations in this parameter could lead to substantial fluctuations in the INMB, establishing it as the primary factor influencing the economic conclusion regarding universal screening. Other parameters with a considerable impact on the results included surgical costs, the probability of progression from mild to moderate severity (per 3-month cycle), and the probability of progression from moderate to severe severity (without brace/with brace). In contrast, parameters such as brace cost, brace initiation rate, and the utility value for the severe health state had a relatively minor impact on the INMB and did not alter the overall conclusion from the base-case analysis that the universal screening strategy remained the dominant option.

**Figure 4:**
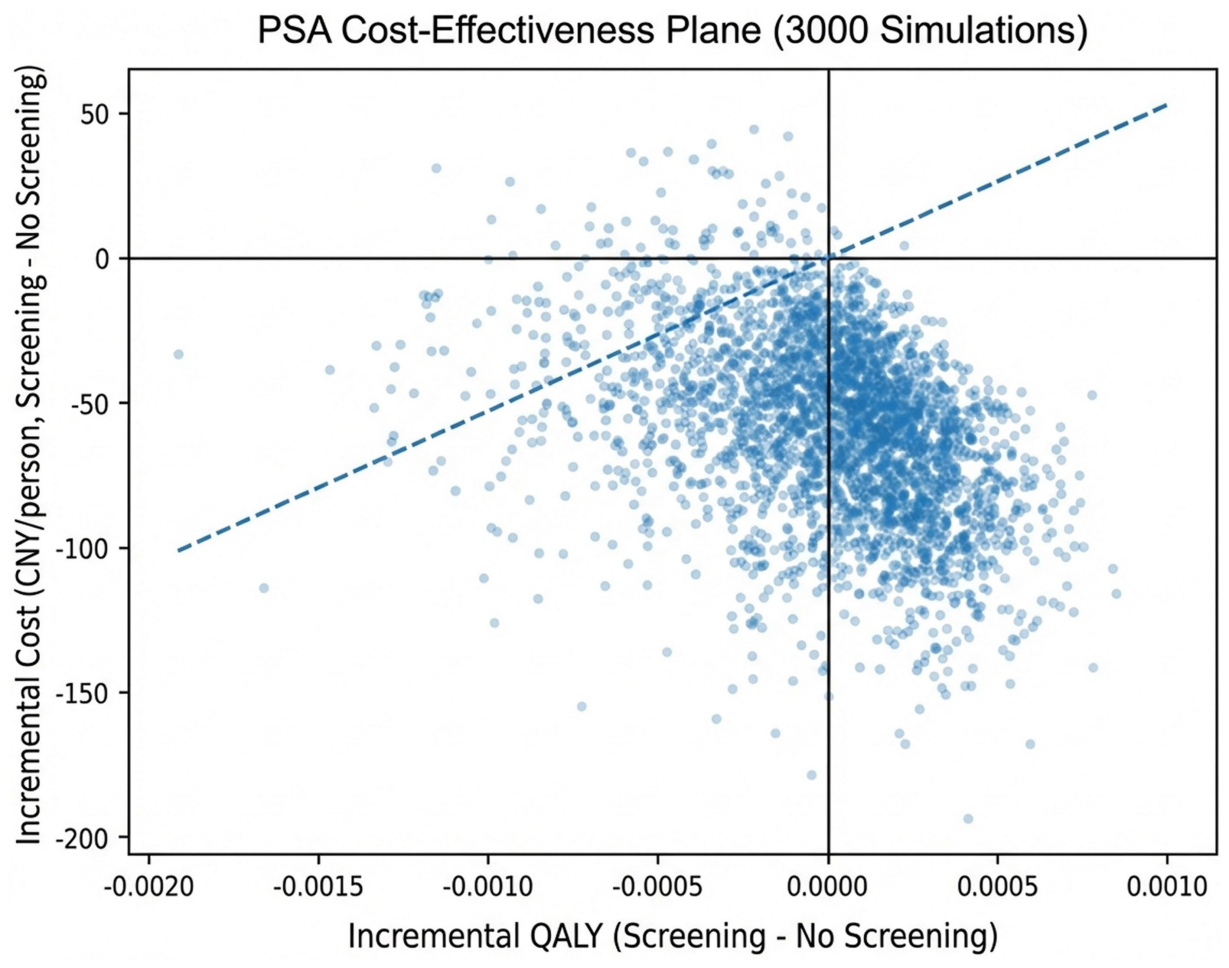
Probabilistic sensitivity analysis: cost-effectiveness plane.

**Figure 5:**
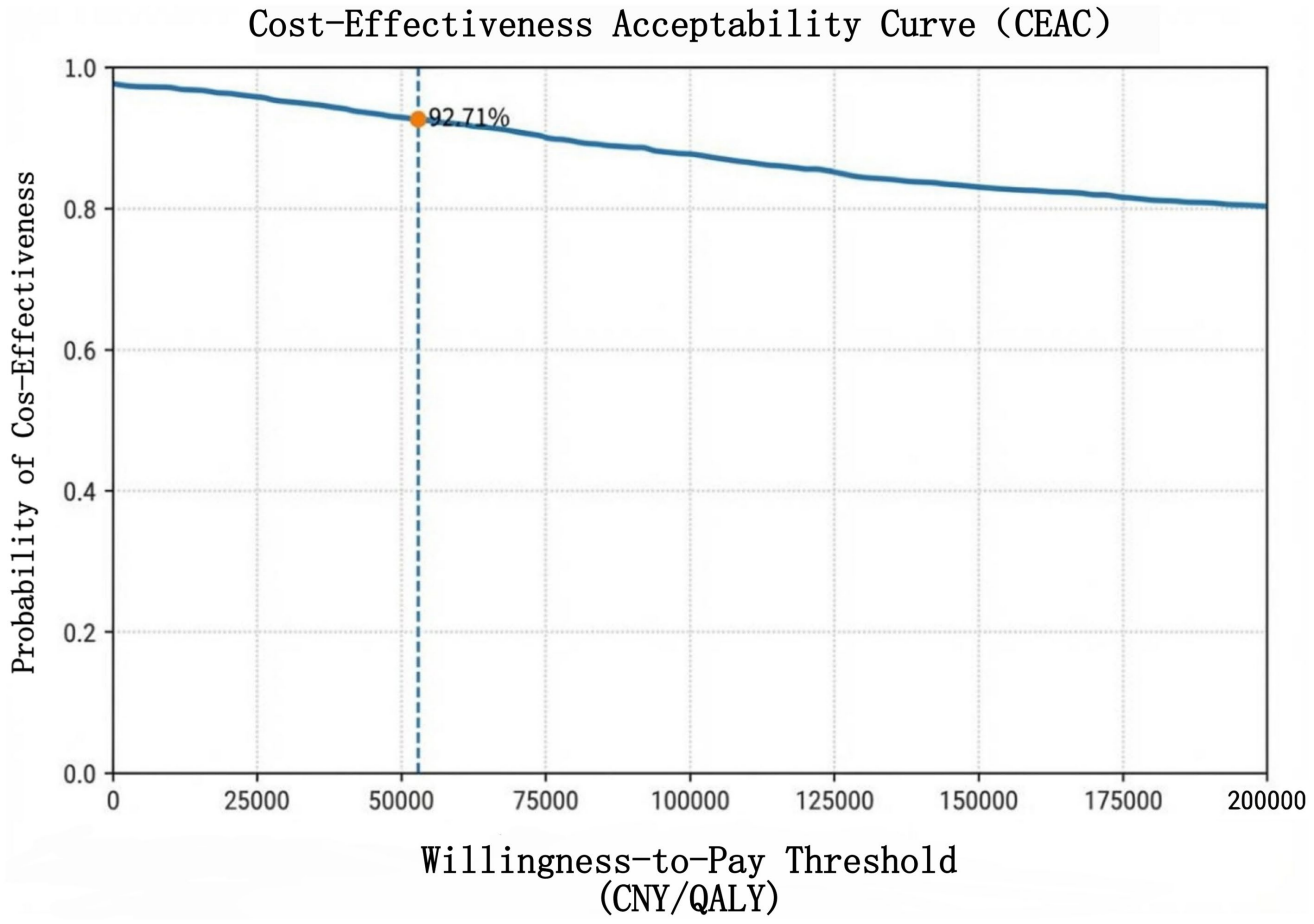
Cost-effectiveness acceptability curve. Dashed line: 1 × GDP per capita (5,285 CNY/QALY).

A probabilistic sensitivity analysis involving 3,000 Monte Carlo simulations was conducted, accounting for the joint uncertainty of all parameters (Figure 4). The cost–utility plane revealed that the majority of simulated points were located in the cost-saving region (ΔCost < 0), while the corresponding incremental QALYs were generally close to zero. This suggests that the universal screening strategy is cost-saving but yields limited health utility gains in most scenarios. At a willingness-to-pay (WTP) threshold of 52,825 CNY/QALY, the universal screening strategy demonstrated a high probability of being cost-effective under joint parameter uncertainty. The cost–utility acceptability curve (CEAC; Figure 5) further indicated that the probability of the universal screening strategy being considered cost-effective progressively increased with a rising WTP threshold.

## Discussion

At a willingness-to-pay (WTP) threshold of 52,825 CNY per QALY (one time the per capita GDP of Gansu Province), the school-based screening strategy was dominant compared to opportunistic detection. The screening strategy yielded a discounted per-person cost of US$18.03 and 3.655057 QALYs, while the no-screening strategy resulted in a discounted per-person cost of US$26.18 and 3.655052 QALYs. This translated to an incremental cost saving of US$8.15 per person and a minimal QALY gain of 0.000005, producing a positive incremental net monetary benefit (INMB) of US$8.19 per person.

The economic advantage of screening arises primarily from cost offsets rather than substantial short-term health gains. The minimal QALY difference between strategies is expected, given both the limited time horizon and the relatively small utility differences between health states for mild and moderate AIS during adolescence (43, 44). Crucially, early case identification through screening may prevent some patients from progressing to severe curves requiring surgery, thereby averting high-cost treatment. This aligns with the economic rationale for population screening: when screening costs are low and the intervention can prevent expensive downstream clinical events, screening may remain economically favorable even with limited incremental health gains within the model’s timeframe (45). Consequently, the economic appeal of screening depends largely on the balance between its unit cost and the number of severe cases it prevents. If screening can cover a sufficiently large population at low cost and effectively reduce the surgical rate, it can achieve net savings even without major gains in health utility (12). Furthermore, long-term follow-up could reveal greater health benefits, further enhancing cost-effectiveness.

Sensitivity analyses show that the economic conclusion is most sensitive to assumptions regarding the efficacy of the opportunistic detection pathway—specifically, the proportion of AIS cases that remain undetected or experience delayed diagnosis in the absence of organized screening (46). This reflects local environmental factors, including disease awareness, access to specialized services, and healthcare-seeking behaviors. In regions with weaker routine detection capacity, the value of screening increases substantially, as it enables earlier entry into management and reduces the likelihood of progression to surgery (12). Conversely, where opportunistic detection is relatively effective, the incremental value of formal screening declines (47). Other influential parameters include surgical costs and the probabilities of progression from mild to moderate and from moderate to severe curvature, further confirming that the economic signal from screening stems mainly from its capacity to alter the disease trajectory away from high-cost, severe states.

Compared to studies by Maciej et al. (12, 48), our findings reinforce the importance of context. Previous analyses in high-income settings have often questioned the cost-effectiveness of universal AIS screening (49), citing high operational costs and limited clinical benefits within healthcare systems that possess some opportunistic detection capacity (48). In contrast, this evaluation, conducted in a resource-limited region with potentially insufficient routine diagnostic capacity, suggests that low-cost screening can be a cost-saving strategy. This discrepancy underscores the necessity of localizing health economic evaluations and cautions against directly extrapolating conclusions across different health systems and economic environments.

Probabilistic sensitivity analysis further supports the robustness of the baseline results. As in previous studies (50, 51), most simulated iterations clustered in the “cost-saving” quadrant of the cost-effectiveness plane, with incremental QALYs near zero. This pattern indicates that, across a wide range of plausible parameter combinations, school-based screening tends to be cost-saving during adolescence but does not confer significant QALY gains. This should not be misinterpreted as a lack of clinical value; rather, it suggests the primary economic benefit of screening derives from averting high-cost events like surgery, while important quality-of-life benefits related to body image, psychological health, and long-term functional outcomes—which may manifest in adulthood—are not fully captured in this time-limited model (52, 53).

From an implementation perspective, this study emphasizes that screening should be viewed as a continuous process encompassing screening, diagnosis, and treatment management, rather than a one-time school check. The most modifiable factor impacting economic value is likely the completion of radiological confirmation and initiation of appropriate management—particularly brace treatment for moderate cases—among screen-positive individuals. In the actual program, a significant bottleneck was the low proportion (27.7%) of secondary screening-positive individuals who completed radiological confirmation, which substantially diminished potential health gains. Implementation strategies to reduce barriers to confirmation (54), such as optimizing referral pathways, coordinating appointments, providing logistical and financial support, establishing school-hospital collaborations, and conducting family-oriented health education, could shift screening from being primarily cost-saving toward generating more quantifiable QALY gains. This would increase the probability of achieving acceptable cost-effectiveness under a broader range of parameter assumptions. Improving adherence to brace treatment among diagnosed moderate cases would further amplify clinical and economic benefits (44, 55).

The significance of this study is multifaceted. First, the use of real-world screening data from Gannan Tibetan Autonomous Prefecture enhances the local relevance of the findings, offering actionable insights for policy-making in resource-limited regions. Second, the integration of a decision tree and Markov model provides a comprehensive framework for economic evaluation, balancing cost and health outcomes. Third, the scenario analysis strengthens the evidence base, demonstrating that the conclusion of school screening being cost-saving is robust under varying assumptions about diagnostic capacity.

However, this study also has limitations. First, some natural history and utility parameters were drawn from external literature and adapted to the 3-month cycle via standard rate-to-probability conversions; although adjustments and calibration were performed where possible, regional variations in disease behavior or health preferences may affect their accuracy. Second, the model did not explicitly capture dynamic factors such as time-varying brace adherence, heterogeneity across different ages or skeletal maturity levels, or potential psychosocial harms from screening and labeling. Third, utility values were primarily derived from previously published EQ-5D-based estimates rather than local preference weights, and generic instruments may underestimate the impact of AIS on body image and psychosocial well-being. Fourth, the analysis adopted a healthcare system perspective and did not incorporate indirect costs such as caregiver time, productivity losses, or long-term disability; for an intervention that can prevent severe deformity and repeated healthcare utilization, this may lead to a conservative estimate of its economic value.

In summary, within the current model time horizon and implementation conditions, school-based adolescent scoliosis screening in Gannan Tibetan Autonomous Prefecture may reduce overall costs at the healthcare system level while providing a slight short-term QALY gain, resulting in a favorable INMB in the baseline scenario. Its economic attractiveness depends heavily on local diagnostic capacity in the absence of screening and on parameters influencing surgical rates. Future research should prioritize obtaining local longitudinal disease progression data to accurately characterize progression and detection pathways, evaluate interventions to improve the linkage between screening and diagnosis, and extend the model time horizon to capture long-term adult outcomes. Such evidence will help optimize screening strategies and strengthen their role within comprehensive adolescent spinal health planning in resource-limited settings.

## Conclusion

This study suggests that universal school-based screening for AIS could be cost-saving. Early detection and intervention may decrease the proportion of cases that advance to severe stages necessitating surgery, which would reduce overall healthcare costs. This conclusion, however, depends on parameters including the baseline case detection rate and disease progression rates, highlighting its context-specific nature. Future work should integrate local long-term follow-up data to refine screening strategies and establish a more robust economic rationale for AIS prevention and control.

## Data Availability

The original contributions presented in the study are included in the article/supplementary material, further inquiries can be directed to the corresponding authors.
